# MicroRNA-891b is an independent prognostic factor of pancreatic cancer by targeting Cbl-b to suppress the growth of pancreatic cancer cells

**DOI:** 10.18632/oncotarget.11001

**Published:** 2016-08-02

**Authors:** Qian Dong, Ce Li, Xiaofang Che, Jinglei Qu, Yibo Fan, Xiaohan Li, Yue Li, Qian Wang, Yunpeng Liu, Xianghong Yang, Xiujuan Qu

**Affiliations:** ^1^ Department of Oncology, Shengjing Hospital of China Medical University, Shenyang, 110004, China; ^2^ Department of Medical Oncology, The First Hospital of China Medical University, Shenyang, 110001, China; ^3^ Department of Pathology, Shengjing Hospital of China Medical University, Shenyang, 110004, China; ^4^ Department of Medical Oncology, The Liaoning Provincial Tumor Hospital, Shenyang, 110042, China

**Keywords:** pancreatic ductal adenocarcinoma (PDAC), microRNA-891b (miR-891b), Cbl-b, prognosis, overall survival (OS)

## Abstract

Growing evidence has revealed that microRNAs could regulate the proliferation of pancreatic ductal adenocarcinoma (PDAC) cells and predict the prognosis of PDAC. Here the comparative microRNA expression profiles of the good and poor prognosis groups were performed by microRNA microarray. MicroRNA-891b (miR-891b) was screened and validated to be a prognostic predictor of PDAC in the initial group and further evaluated to be an independent predictor for the overall survival of resectable PDACs in an independent cohort. By a series of cellular and animal experiments, as well as clinical specimen analyses, miR-891b was confirmed to target the Cbl-b gene, promot the expression of tumor suppressor p21 protein and inhibit the proliferation of PDAC cells. The results provide a theoretical basis for the study of miR-891b as an independent prognostic predictor of PDAC and the role of miR-891b/Cbl-b pathway in this prediction, as well as the identification of new targets for PDAC.

## INTRODUCTION

Pancreatic cancer, which is the seventh most common cause of cancer death worldwide [1], has a very poor prognosis and the 5-year survival rate is < 5% [2]. In China, the 5-year survival rate in patients with pancreatic cancer is 4.1% and the median survival time is only 3.9 months [3]. Less than 20% patients with pancreatic cancer are candidates for radical surgery [4]; however, the 5-year survival rate of these patients is only 10%–25% [5]. Pancreatic cancer is a hypovascular tumor. Tumor cells can survive under conditions of low blood supply or hypoxia, which suggests that pancreatic cancer cells have a very strong proliferative capacity [6]. This characteristic of pancreatic cancer significantly affects the prognosis of patients. Though a series of new agents, including Nab-paclitaxel and inhibitors of MEK, PI3K, and mTOR, have been introduced, only the combination of erlotinib plus gemcitabin can achieve a 4-week overall survival (OS) benefit for patients [7]. Therefore, identification of novel and effective targets for pancreatic cancer is an urgent issue.

MicroRNA (miRNA) is a widely-distributed, single-stranded, non-coding RNA molecule, which is involved in the functional regulation of target genes by binding to the 3′-untranslational region (UTR) of target genes and degrading or inhibiting the translation of mRNA [8]. MiRNA is shorter than mRNA and has a stronger tolerance for the degradation of ribonuclease. MiRNAs in formalin-fixed paraffin-embedded specimens (FFPEs) and body fluid can be fully preserved, which have been discovered to detect and predict prognosis in patients with cancer [9, 10]. Lin *et al.* used array analysis and reverse transcription-quantitative real-time polymerase chain reaction (qRT-PCR) to build a serum miRNA classifier (containing miR-29a, miR-29c, miR-133a, miR-143, miR-145, miR-192 and miR-505) to detect hepatocellular carcinoma and can identify different kinds of hepatocellular carcinoma in patients at risk [11]. In addition, Kleivi Sahlberg *et al.* identified a four-miRNA signature (miR-18b, miR-103, miR-107 and miR-652) that predicted tumor relapse and OS for patients with triple-negative breast cancer [12]. Growing evidence has revealed that miRNAs participate in the proliferative regulation of pancreatic cancer cells and influence the prognosis of the disease [13, 14]. However, the studies about the prognostic significance of miRNAs detected by a scientific screening model in pancreatic ductal adenocarcinoma (PDAC) are less reported.

In this study, the patients with similar clinicopathologic features and treatment but completely different outcomes composed the initial screening cohort. These patients were divided into a good prognosis group and a poor prognosis group (each group had 10 cases). We used the miRNA expression chips to detect the miRNA expression profiles of the cases in these two groups. It was screened and validated that the expression of miR-891b was significantly different between the two groups. Moreover, miR-891b was evaluated to be an independent predictive factor for the OS of resectable PDAC patients in an independent cohort with a larger sample size (114 cases). We further performed cellular and animal experiments, as well as clinical specimen analyses, to confirm that miR-891b could inhibit the proliferation of PDAC by promoting the expression of tumor suppressor p21 protein, which was achieved by targeting inhibition of the expression of the Cbl-b gene.

## RESULTS

### Screening and validation of miR-891b as a prognostic predictor for PDAC

**Figure 1:**
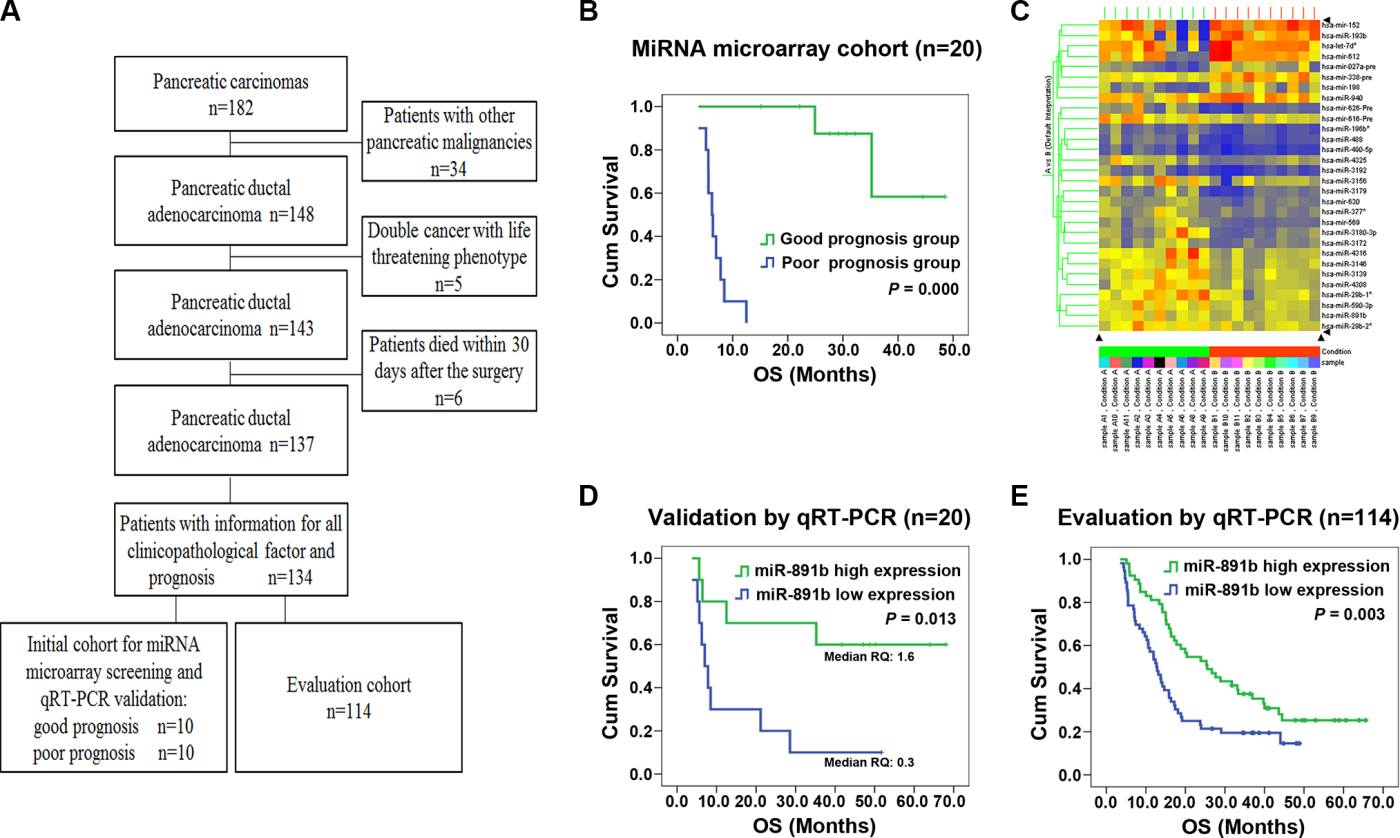
Screening, validation and evaluation of miR-891b as a prognostic predictor for PDAC (**A**) The flowchart of patient selection and schematic design. (**B**) Comparisons of overall survival (OS) between the good and the poor prognosis groups in the initial screening cohort. The patients in the good prognosis group had a median OS of 48.0 months compared with 6.3 months for patients in the poor prognosis group. Significant differences in survival were revealed by log rank test (log rank *x*^2^ = 21.837, *P* = 0.000). (**C**) The heatmap of 30 miRNAs differentially expressed between the good and poor prognosis groups using miRNA microarray. (**D–E**) Kaplan-Meier curve for patients with pancreatic ductal adenocarcinoma (PDAC) and different levels of expression of miR-891b in miRNA microarray cohort and miRNA evaluation cohort using qRT-PCR. (D) In miRNA microarray cohort, PDAC patients with high or low levels of expression of miR-891b had a median overal survival (OS) time of 44.4 or 7.0 months, respectively (log rank *x*^2^ = 6.155, *P* = 0.013). (E) In miRNA evaluation cohort, PDAC patients with high or low levels of expression of miR-891b had a median OS time of 25.5 or 12.7 months, respectively (log rank *x*^2^ = 8.791, *P* = 0.003).

The flowchart of patient selection and schematic design were shown in Figure 1A. To screen for the miRNAs that can be used as prognostic predictors for patients with resectable PDAC, the miRNA microarray was performed in the good and poor prognosis groups of the initial screening cohort. The patients in the good prognosis group had a median OS of 48.0 months compared with 6.3 months for patients in the poor prognosis group (log rank *x*^2^ = 21.837; *P* = 0.000, Figure 1B). There were no statistically significant relationships for the other clinicopathologic characteristics between the two groups (all *P* > 0.05, Table 1). Thirty miRNAs were identified as being differentially expressed between the good and poor prognosis groups (all *P* < 0.05; Figure 1C) by miRNA microarray analysis. Twenty-two miRNAs were up-regulated and eight were down-regulated in the good prognosis group compared with those in the poor prognosis group. These differentially-expressed miRNAs were found to have tumor-associated putative target genes. Among them, we have drawn more attention to miR-891b, which has not been previously described in PDAC. The expression of miR-891b was screened to be up- regulated in the cases of the good prognosis group by the miRNA microarray. Furthermore, the expression of miR-891b was detected in these 20 cases of the initial cohort by using qRT-PCR to validate the prognostic value. The median relative quantitation of miR-891b (0.7) was used as the cut-off point to categorize the patients. Patients with high or low levels of expression of miR-891b had a median OS of 44.4 or 7.0 months, respectively (log rank *x*^2^ = 6.155, *P* = 0.013; Figure 1D). A strong correlation between miR-891b expression status and OS was demonstrated, confirming that miR-891b was a prognosis predictor for PDAC.

**Table 1:** The demographic and clinicopathologic characteristics of the patients with pancreatic ductal adenocarcinoma treated by surgical resection

Characteristics	Patients for the miRNA microarray			Patients of the evaluation cohort (*n*)
	Good prognosis group	Poor prognosis group	*P* value	
No. Patients	10	10		114
Age (years) < 60	4	6	0.371	55 (48.2%)
≥ 60	6	4		59 (51.8%)
Gender Male	4	6	0.371	68 (59.6%)
Female	6	4		46 (40.4%)
Location of tumor Head	8	9	0.500	88 (77.2%)
Body or tail	2	1		26 (22.8%)
Type of operation Pancreaticoduodenectomy	8	8	0.709	88 (77.2%)
Distal pancreatectomy	2	2		25 (21.9%)
Total pancreatectomy	0	0		1 (0.9%)
Maximum tumor diameter (mean ± SD) (cm)	4.35 ± 1.23	4.75 ± 1.44	0.512	4.22 ± 1.81
Differenciation Well	4	3	0.881	40 (35.1%)
Moderately	4	5		57 (50.0%)
Poor	2	2		17 (14.9%)
Surgical margins Negative	9	10	0.500	111 (97.4%)
Positive	1	0		3 (2.6%)
pT category pT1+ pT2	4	3	0.500	58 (50.9%)
pT3+pT4	6	7		56 (49.1%)
pN category pN0	4	4	0.675	84 (73.7%)
pN1	6	6		30 (26.3%)
Vessel invasion No	5	3	0.325	61 (53.5%)
Yes	5	7		53 (46.5%)
Vascular tumor thrombus No	10	10	---	111 (97.4%)
Yes	0	0		3 (2.6%)
Adjacent organs invasion No	7	8	0.500	86 (75.4%)
Yes	3	2		28 (24.6%)
pTNM category I	0	0	0.675	53 (46.4%)
IIA	4	4		15 (13.2%)
IIB	6	6		18 (15.8%)
III	0	0		28 (24.6%)
CA19-9 (median, range) (U/mL)	250.0 (9.69–739.70)	130.40 (0.6–1551)	0.791	287.61 (0.60–12076.00)

### Evaluation of miR-891b as an independent prognostic factor for patients with resectable PDAC

To evaluate the prognostic role of miR-891b for patients with resectable PDAC, the expression of miR-891b was detected in 114 independent PDAC samples by qRT-PCR. This evaluation cohort contained all of the resectable cases, including stage I, II, and III tumors. The other clinicopathologic characteristics did not differ significantly compared to the initial cohort of patients (Table 1). The expression of miR-891b was detectable in all of the cases. The median relative quantitation of miR-891b (0.6) was used as the cut-off point to categorize the patients. A strong correlation between miR-891b expression status and OS was demonstrated. Patients with high miR-891b expression had a signifiantly longer median OS (25.5 months; 95% CI, 16.4–34.6 months) compared to the patients with low miR-891b expression (median OS, 12.7; 95% CI, 10.5–14.9 months; log rank *x*^2^ = 8.791, *P* = 0.003; Figure 1E). In the multivariate Cox proportional hazards model (forward) analysis, miR-891b high expression (HR, 0.467; 95% CI, 0.287–0.759; *P* = 0.002), age ≥ 60 years (HR, 0.612; 95% CI, 0.376–0.995; *P* = 0.048), serum CA19-9 level ≥ 37 U/mL (HR, 4.073; 95% CI, 1.728–9.601; *P* = 0.001), and poorly differentiated tumor (HR, 2.951; 95% CI, 1.481–5.878; *P* = 0.002) were significant independent prognostic factors associated with OS (Table 2). These data suggest that miR- 891b acts as an independent prognostic factor in patients with resectable PDAC.

**Table 2:** Significant independent prognostic factors for overall survival in patients with pancreatic ductal adenocarcinoma treated by surgical resection on multivariate analysis

Characteristics	Category	Hazard Ratio	95% CI	*P* value
miR-891b expression	Low vs. high	0.467	0.287-0.759	0.002
Age (years)	< 60 vs. ≥ 60	0.612	0.376-0.995	0.048
CA19-9 (U/mL)	< 37 vs. ≥ 37	4.073	1.728-9.601	0.001
Differenciation	well	1		
	moderately	1.918	1.102-3.339	0.021
	poorly	2.951	1.481-5.878	0.002

* The multivariate Cox proportional hazards model (forward) was fitted using all of the clinical and pathological variables, which included age (< 60 vs. ≥ 60 years old), gender (male vs. female), location of tumor (head vs. body or tail), type of operation (pancreaticoduodenectomy vs. distal pancreatectomy vs. total pancreatectomy), maximal tumor diameter, histological differentiation (well vs. moderately vs. poorly differentiated), surgical margins (negative vs. positive), pT category (pT1+pT2 vs. pT3+pT4), pN category (pN0 vs. pN1), vessel invasion (no vs. yes), vascular tumor thrombus (no vs. yes), adjacent organs invasion (no vs. yes), pTNM category (I vs. II vs. III),miR-891b expression (low expression vs. high expression), and CA19-9 level (< 37 U/mL vs. ≥ 37 U/mL).

### Relationship of the miR-891b levels to the clinicopathologic characteristics of patients with resectable PDAC

The relationship between miR-891b expression and the clinicopathologic characteristics were compared. As shown in Table 3, the miR-891b levels were strongly associated with tumor diameter, pT, pN, and the pTNM stage (*P* < 0.05). There were no statistically significant relationships between miR-891b expression and the other clinicopathologic characteristics (all *P* > 0.05). Patients with miR-891b high expresssion was more common with small tumor, T1/T2, N0, and early TNM stage. These data support the notion that miR-891b may act as a tumor suppressor in PDAC.

**Table 3:** Relationship between the expression of miR-891b and clinicopathologic characteristics in patients with pancreatic ductal adenocarcinoma patients treated by surgical resection

Characteristics	miR-891b high expression (*n*,%)	miR-891b low expression (*n*,%)	*P* value
Age (years) < 60	27(48.2)	28(48.3)	0.995
≥ 60	29(51.8)	30(51.7)	
Gender Male	37(66.1)	31(53.4)	0.170
Female	19(33.9)	27(46.6)	
Location of tumor Head	46(82.1)	42(72.4)	0.216
Body or tail	10(17.9)	16(27.6)	
Type of operation Pancreaticoduodenectomy	47(83.9)	41(70.7)	0.189
Distal pancreatectomy	9(16.1)	16(27.6)	
Total pancreatectomy	0(0)	1(1.7)	
Maximum tumor diameter (cm) (mean ± SD)	3.83 ± 1.50	4.58 ± 2.00	0.027
Differenciation Well	19(33.9)	21(36.2)	0.373
Moderately	31(55.4)	26(44.8)	
Poor	6(10.7)	11(19.0)	
Surgical margins Negative	53(94.6)	58(100)	0.115
Positive	3(5.4)	0(0)	
pT category pT1+ pT2	34(60.7)	24(41.4)	0.039
pT3+pT4	22(39.3)	34(58.6)	
pN category pN0	51(91.1)	33(56.9)	0.000
pN1	5(8.9)	25(43.1)	
Vessel invasion No	34(60.7)	27(46.6)	0.130
Yes	22(39.3)	31(53.4)	
Vascular tumor thrombus No	56(100.0)	55(94.8)	0.244
Yes	0(0.0)	3(5.2)	
Adjacent organs invasion No	44(78.6)	42(72.4)	0.445
Yes	12(21.4)	16(27.6)	
pTNM category I	33(58.9)	20(34.5)	0.015
II	10(17.9)	23(39.7)	
III	13(23.2)	15(25.9)	
CA19-9 (U/mL) < 37.0	7(14.9)	8(15.1)	0.978
≥ 37.0	40(85.1)	45(84.9)	

Abbreviations: pT: pathologic T; pN: pathologic N; pTNM: pathologic TNM.

### MiR-891b inhibited proliferation of pancreatic cancer *in vitro* and *in vivo*

**Figure 2:**
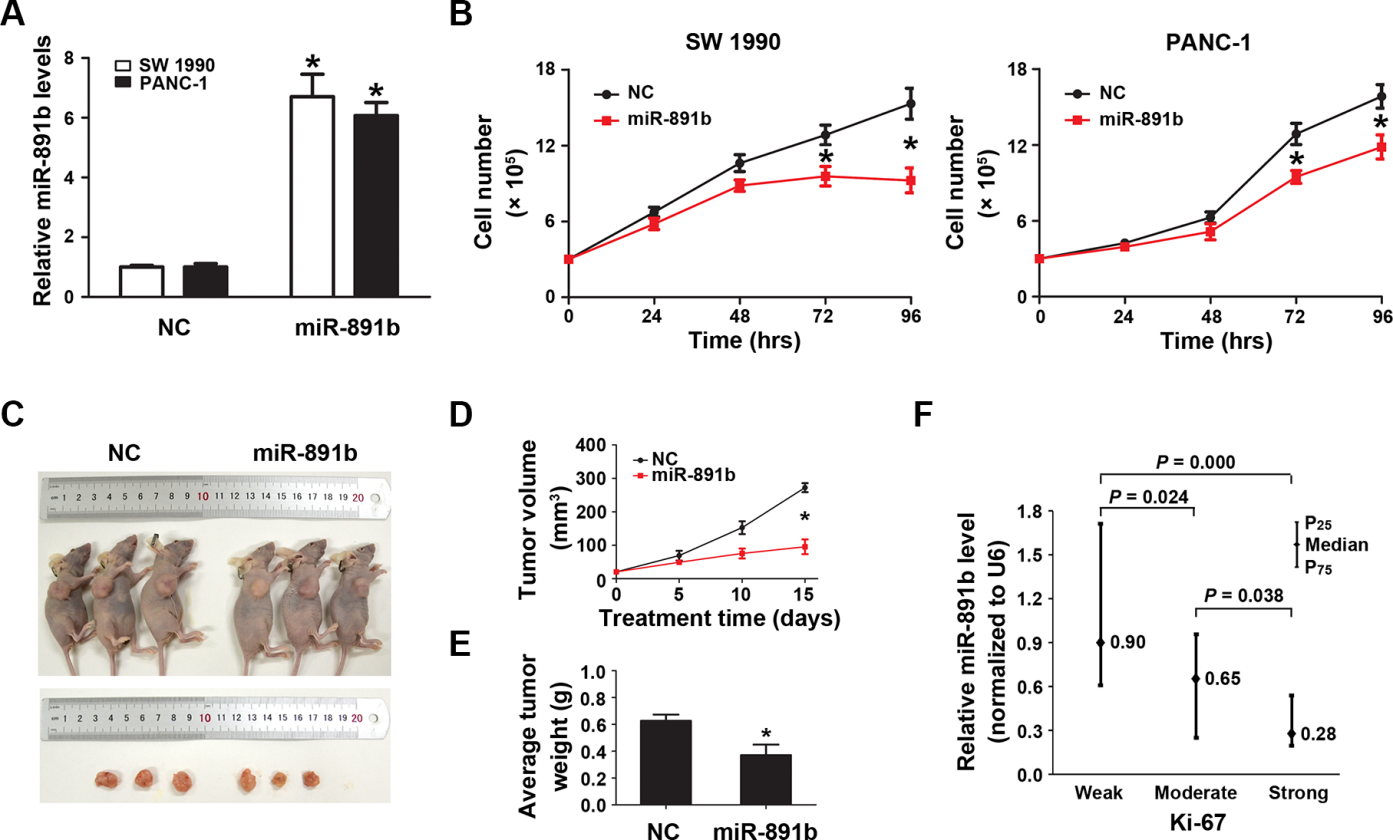
Overexpression of miR-891b significantly inhibited the proliferative capacity of PDAC cells (**A**) Following miR-891b transfection fold changes in the miR-891b levels were determined through qRT-PCR. U6 small nuclear RNA was used as an internal control for relative quantitation. *P < 0.05. (**B**) PDAC cell lines, SW1990 and PANC-1, were transfected with miR-891b mimic or NC. Cells were collected 24, 48, 72, and 96 h after the transfection. The proliferation curve was drawn by using the trypan blue cell counting method. The results suggested miR-891b significantly inhibited the proliferation of PDAC cells (mean ± SD, results of three independent experiments, *P < 0.05). (**C**) Establishment of a nude mouse model of PDAC subcutaneous transplantation tumor with SW1990 cell line. MiR-891b agomir was intratumorally injected after the tumor was formed. After 2 weeks, compared with NC-treated tumor, the size of the subcutaneous tumor treated with miR-891b agomir significantly decreased. (**D**) The volume of the subcutaneous tumor was measured regularly during the period of the injection to draw the growth curve of the tumor. The results suggested that compared with NC-treated tumors the volume of miR-891b agomir-treated tumors decreased significantly. The error line represents the mean ± SD, *P < 0.05. (**E**) The nude mice were killed after 2 weeks of treatment. The tumors were completely dissected and weighted. The weight of the tumor treated with miR-891b agomir decreased significantly compared with tumor treated with NC. The error line represents the mean ± SD, *P < 0.05. (**F**) The expression of Ki-67 protein in human PDAC tissue samples was detected using an immunohistochemical assay. The expression of miR-891b was evaluated by qRT-PCR. The relative quantitation of miR-891b was expressed as medians with interquartile (P_25_–P_75_). The comparisons between the expression of miR-891b and Ki-67 protein were performed with a Mann-Whitney *U* test. The results showed that the expression of miR-891b significantly varied with the expression of Ki-67.

To determine the impact of miR-891b on PDAC viability, we altered the expression of miR-891b in SW1990 and PANC-1 cells by transfecting the cells with miR-891b mimic. We observed a several fold increase in the miR-891b levels following transient transfection by qRT-PCR (Figure 2A). Up-regulated expression of miR-891b significantly suppressed tumor cell growth (Figure 2B). The anti-tumor effect of miR- 891b was confirmed by *in vivo* experiments using a pancreatic cancer xenograft mouse model. Intratumoral miR-891b agomir was administered in established tumors. The tumor volume and weight regressed with miR-891b replenishment compared to negative contorl (NC)-treated mice that showed a gradual increase in tumor volume over time (Figure 2C–2E). What's more, we found that the levels of miR-891b were negatively related with the expression of Ki-67 (Spearman correlation coefficient: −0.359, *P* = 0.000) by clinical specimen analyses. It was demonstrated that the weak levels of expression of Ki-67 in PDAC tissues, while having high miR-891b expression (Figure 2F). While, the expression of Ki-67 was observed to be very strong in cases with low expression of miR-891b (Figure 2F). These experimental and clinical evidences strongly support the notion that miR-891b is a suppressor of PDAC proliferation.

### MiR-891b directly binds to the 3′UTR of human Cbl-b

**Figure 3:**
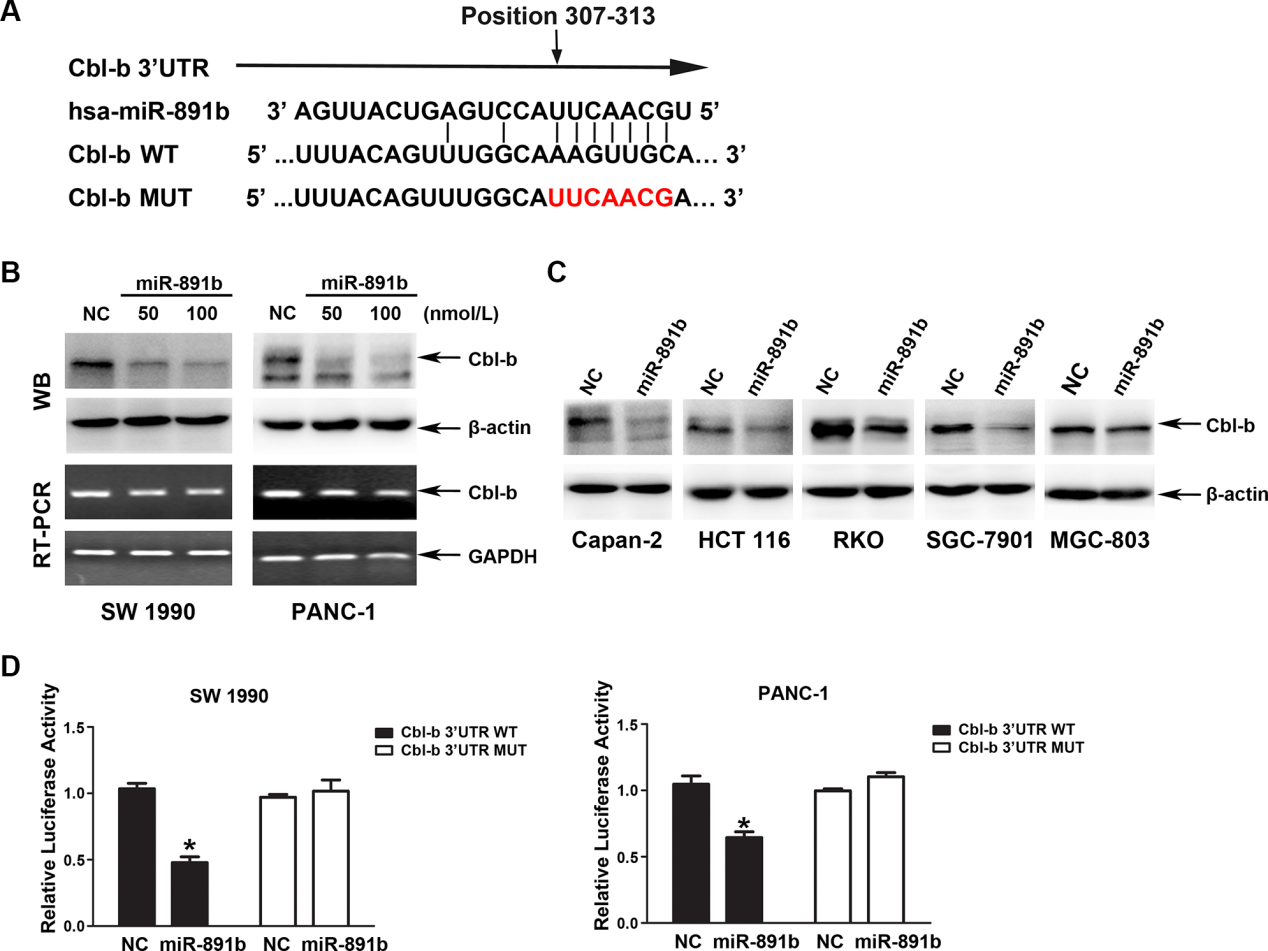
Cbl-b is a direct target gene of miR-891b (**A**) The bindng site of miR-891b to Cbl-b 3′UTR and construction of plasmids containing Cbl-b 3′UTR sequence or binding site-mutated sequence. (**B**) miR-891b inhibited the expression of Cbl-b at the post-transcriptional level. PDAC cell lines, SW1990 and PANC-1, were transfected with miR-891b mimic in different concentrations. Western blot indicated miR-891b down-regulated the expression of Cbl-b protein. RT-PCR suggested overexpression of miR-891b did not significantly affect the level of Cbl-b mRNA. (**C**) The pancreatic cancer cell line, Capan-2, and the human colon cancer cell lines, HCT 116 and RKO, as well as the human gastric cancer cell lines, SGC-7901 and MGC-803, were transfected with miR-891b mimic or NC. Western blot showed that overexpression of miR-891b significantly down-regulated the level of Cbl-b protein in the above cell lines. (**D**) The PDAC cell lines, SW1990 and PANC-1, were co-transfected with pMirTarget-Cbl-b WT (or MUT) plasmid, miR-891b mimic (or NC), and pRL-TK. The dual luciferase reporter assay detected the activity of luciferase. The histogram shows the relative activity of luciferase (mean ± SD, results of three independent experiments, *P < 0.05). The results suggest that miR-891b directly binds to the 3′UTR of Cbl-b.

To fully understand the mechanisms by which miR- 891b executes its function, we adopted two bioinformatic algorithms (TargetScan and microRNA.org) to identify a large number of potential target genes of miR-891b, such as epidermal growth factor receptor (EGFR), Caveolin-1, Bcl-2, c-Cbl and Cbl-b, etc. Among these candidates, only the protein expression level of Cbl-b was obviously suppressed after overexpression miR-891b (data not shown). Thus, Cbl-b was one of the most likely target genes for miR-891b. A putative seed sequence of miR-891b was observed in the 3′UTR of the Cbl-b transcript (Figure 3A), which was experimentally tested in SW1990 and PANC-1 cells via transient transfection of miR-891b mimic or NC. Our data revealed a significant dose-dependent down-regulation of Cbl-b at the protein level, but no apparent change at the transcript level in miR-891b mimic-transfected cells (Figure 3B). We observed that miR-891b generally suppressed Cbl-b protein expression in other types of human cancer cells, including colorectal and gastric cancer cells (Figure 3C). These data suggest that miR-891b down-regulates Cbl-b expression via a post-transcriptional mechanism.

To validate the ability of miR-891b to target Cbl-b, we used the dual luciferase reporter assay. We co-transfected the SW1990 and PANC-1 cells with miR-891b mimic or NC and a firefly luciferase reporter plasmid containing a region of full-length 3′UTR of Cbl-b mRNA harboring the miR-891b target site (position 307–313). As a control, Cbl-b 3′UTR mutated vector was constructed and the specific sites targeted by miR-891b were mutated. The TK vector expressing renilla luciferase was co-tranfected into cells to normalize the tranfection efficiency. The luciferase activity was substantially decreased in cells transfected with miR-891b mimic compared to NC. Cells transfected with 3′UTR MUT were resistant to the suppressor activity of miR-891b (Figure 3D), suggesting that miR-891b negatively regulates the expression of Cbl-b by directly targeting 3′UTR of the Cbl-b transcript.

### Silencing of Cbl-b expression inhibited proliferation in PDAC cells

**Figure 4:**
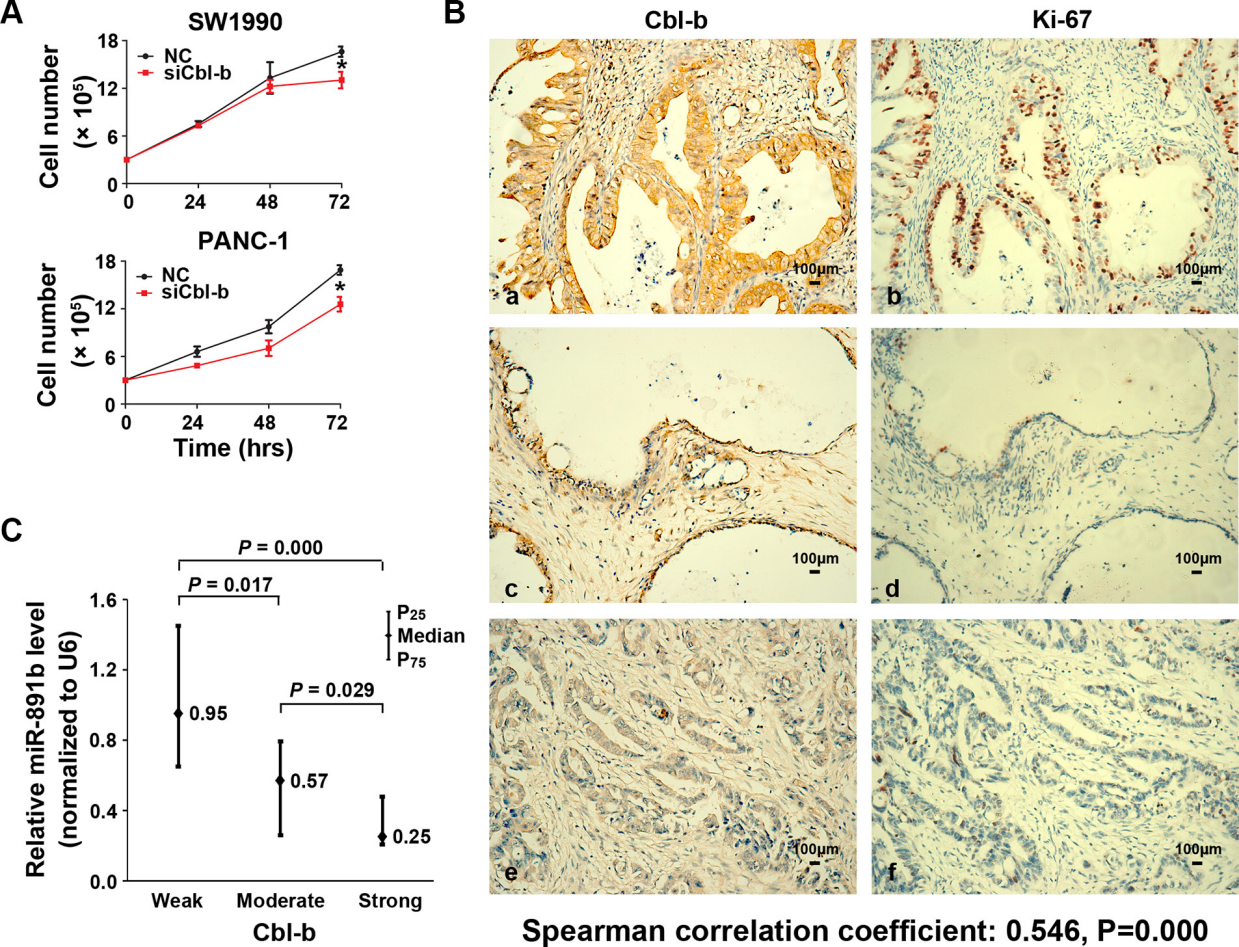
The expression of Cbl-b was associated with the proliferative capacity of PDAC cells (**A**) Silencing the expression of Cbl-b significantly inhibited the proliferative capacity of PDAC cells. The PDAC cell lines, SW1990 and PANC-1, were transfected with Cbl-b siRNA or NC. Cells were collected 24, 48, and 72 h after transfection. The proliferation curve was drawn using the trypan blue cell counting method. The results suggested that silence of Cbl-b significantly inhibits the proliferation of PDAC cells (mean ± SD, results of three independent experiments, *P < 0.05). (**B**) The expression of Cbl-b and Ki-67 in PDAC tissues was detected using immunohistochemistry. a, c and e: the expression of Cbl-b in PDAC tissue. Positive expression shows brown-yellow particles distributed in the membrane and cytoplasm of the cells (SP staining × 200). b, d and f: the expression of Ki-67 in PDAC tissue was detected. Positive expression shows brown-yellow particles distributed in the nuclei of the cells (SP staining × 200). (**C**) The expression of Cbl-b protein in human PDAC tissue samples was detected using an immunohistochemical assay. The expression of miR-891b was evaluated by qRT-PCR. The relative quantitation of miR-891b was expressed as medians with interquartile (P_25_–P_75_). The comparisons between the expression of miR-891b and Cbl-b protein were performed with a Mann-Whitney *U* test. The results showed that the expression of miR-891b significantly varied with the expression of Cbl-b.

To determine whether the effect of miR-891b on cell growth was through targeting the Cbl-b gene, we tested the viability of PDAC cells via silencing Cbl-b expression by transfection of small inferfering RNA (siRNA). Down-regulated expression of Cbl-b significantly suppressed tumor cell growth (Figure 4A). To determine the relationship of Cbl-b expression with Ki-67 expression, the 114 cases of PDAC tissues in the evaluation cohort were used to detected the proteins expression by immunohistochemistry (IHC). We showed that the positive relationship between the expression of Cbl-b protein and nuclear Ki-67 antigen was statistically signficant (Spearman correlation coefficient: 0.546, *P* = 0.000). It was demonstrated that no or faint expression of Ki-67 in PDAC tissues, while having weak expression of Cbl-b (Figure 4B). The Ki-67 levels were observed to be very strong in some tissues, while Cbl-b also had strong expression (Figure 4B). We also found that there was a negative correlation between Cbl-b levels and miR-891b levels in clinical specimen analyses (Figure 4C). The levels of miR-891b were negatively related with the expression of Ki-67 (Spearman correlation coefficient: − 0.419, *P* = 0.000). These data support the notion that the inhibition of proliferation for miR-891b in pancreatic cancer cells was by targeting the Cbl-b gene.

### MiR-891b regulated the expression of Cbl-b-associated tumor suppressor gene

**Figure 5:**
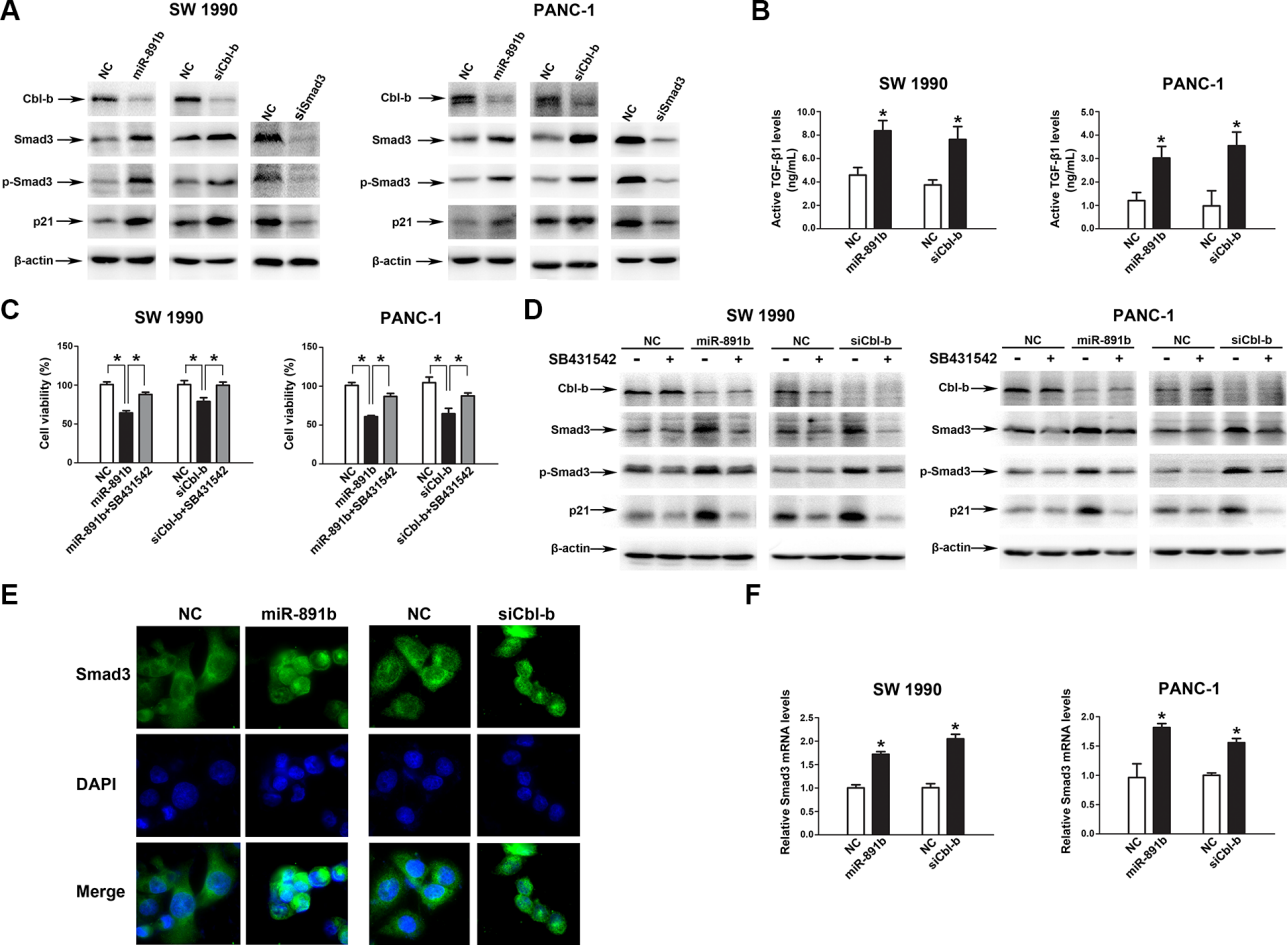
MiR-891b targeted the regulation of the downstream signal molecules of Cbl-b (**A**) MiR-891b was overexpressed, or Cbl-b and Smad3 were silenced in PDAC cell lines, SW1990 and PANC-1. The expressions of associated proteins were detected by western blot. (**B**) The PDAC cells were transfected with miR-891b mimic/NC, or Cbl-b siRNA/NC for 48h, and cell culture supernatants were collected. Active TGF-β1 levels were determined by Elisa. *P < 0.05. (**C**–**D**) After pretreated with or without 10 μmol/L SB431542 for 2 h, PDAC cells were transfected with miR-891b mimic/NC, or Cbl-b siRNA/NC. (C) Cells were collected at 48 h after the transfection. The cell proliferation was determined by counting the non-trypan blue staining cells. *P < 0.05. (D) The expression of Cbl-b, Smad3, p-Smad3 and p21 were detected by western blot analysis. (**E**) After miR-891b mimic/NC or Cbl-b siRNA/NC was transfected in PANC-1 cell line, subcellular localization of Smad3 was identified using immunofluorescence. DAPI, 4′-6-Diamidino-2-phenylindole. (**F**) The PDAC cells were transfected with miR-891b mimic/NC, or Cbl-b siRNA/NC. The Smad3 mRNA level was determined by qRT-PCR. 18S ribosomal RNA was used as an internal control for relative quantitation. *P < 0.05.

Finally, we sought to identify the mechanism underlying the pathway of miR-891b/Cbl-b. A previous study showed that Cbl-b directly binded to Smad3 through a proline-rich motif, thereby preventing Smad3 from interacting with Smad4 and blocking nuclear translocation of Smad3 in breast cancer cells [15]. Silencing of Cbl-b expression resulted in increased expression of TGF-β target genes, such as p21 [15]. To determine whether miR-891b/Cbl-b affected cell proliferation through the Smad3/p21 pathway in PDAC cells, we examined the level of Smad3, p-Smad3, and p21 in SW1990 and PANC-1 cells (Smad4 wild cells) after overexpression miR-891b, silecing Cbl-b or Smad3. The overexpression of miR-891b or silencing Cbl-b resulted in up-regulation of the expression of Smad3, p-Smad3, and p21 (Figure 5A). Silencing the expression of Smad3 down-regulated the expression of p21. The overexpression of miR-891b or Cbl-b knockdown resulted in the enhancement of TGF-β1 levels in supernatants of PDAC cell lines (Figure 5B). Furthermore, SB431542, a potent inhibitor of TGF-β receptor type I, attenuated the growth inhibition which was induced by miR-891b overexpression or Cbl-b knockdown (Figure 5C). Western blot analysis demonstrated that SB431542 inhibited the up-regulated expression of Smad3, p-Smad3 and p21 proteins induced by miR-891b overexpression or Cbl-b knockdown (Figure 5D). Finally, miR-891b overexpression or Cbl-b knockdown increases nuclear translocation of Smad3 without TGF-β1 treatment (Figure 5E). These results indicate that miR-891b overexpression or Cbl-b knockdown active Smad3/p21 pathway by the autocrine effect of TGF-β1 secreted by PDAC cell lines. In addition, we found that miR-891b overexpression or Cbl-b knockdown increased the mRNA level of Smad3 (Figure 5F). Taken together, these results strongly suggest that miR-891b/Cbl-b suppressed cell proliferation through activating the Smad3/p21 pathway in PDAC cells.

## DISCUSSION

A number of studies have shown that miRNAs are involved in the development and progression of pancreatic cancer as oncogenes or tumor suppressor genes and also have potential to be prognostic biomarkers for pancreatic cancer. For example, miR-494 negatively regulates the expression of FOXM1 [11], and miR-145 inhibits the expression of MUC13 [14], to inhibit the proliferation, invasion, and metastasis of pancreatic cancer. MiR-23b is associated with radiotherapy sensitization of the disease mediated by autophagy [16]. MiR-21 [17], miR-211 [18], and miR-1290 [19] are all reported to be independent prognostic factors of pancreatic cancer. In this study, miR-891b was screened to be differentially-expressed in resectable PDAC patients with different prognosis by miRNA microarray analysis and may be used as a novel independent prognostic factor of pancreatic cancer.

MiR-891b is located in the X chromosome, it has been shown that miR-891b may participate in the development and maturation of the epididymis [20]. However, the correlation between miR-891b and the prognosis of resectable PDAC patients, and the mechanisms by which miR-891b facilitates the development of PDAC have not been reported. In the current study, the cases with similar clinicopathologic features and treatment, but having completely different prognosis were included in the screening cohort. A miRNA expression chip was used to test the miRNA expression profiles in these two groups. The differentially-expressed miRNAs in patients with different prognosis were screened. Among them, miR-891b was screened and validated to be an independent prognostic factor for resectable PDAC patients, which suggested that our screening strategy could effectively identify miRNAs with predictive value. Moreover, we explored the correlation between miR-891b and the clinicopathologic characteristics of PDAC, and found the expression of miR-891b to be associated with tumor diameter, pT, pN, and pTNM staging. Furthermore, cellular and animal experiments confirmed the potential of miR-891b in inhibiting the proliferation of PDAC. Thus, the effect of miR-891b on the prognosis of PDAC may be due to the inhibition of the proliferation of PDAC cells.

It is well known that miRNAs participate in regulating the biological behaviors, such as proliferation, differentiation and apoptosis, by regulating the expression of their target genes. However, the functions, target genes, as well as the regulatory mechanism of miR-891b are still unclear in pancreatic cancer. In this study, we used bioinformatics tools, including Targetscan and microRNA.org, to predict the potential target genes of miR-891b and found that Cbl-b was one of the most likely target genes for miR-891b based on preliminary screening. The finding was validated by the dual luciferase reporter gene assay. We have shown that Cbl-b is a direct target gene of miR-891b and the binding site is located at 307-313 bp of 3′UTR of Cbl-b mRNA. Cbl-b consists of the N-terminal tyrosine kinase binding (TKB) domain, linker, RING finger domain, C-terminal proline-rich region, ubiquitin-associated domain, and leucine zipper region [21]. TKB determines the specificity of substrate proteins of Cbl-b by binding to the specific phosphorylated tyrosine residues of substrate proteins. RING finger has intrinsic E3 ligase activity and mediates the transfer of ubiquitin to substrates. Therefore, Cbl-b has dual functions of E3 ubiquitin ligase and adaptor protein [22, 23]. Studies have indicated that Cbl-b promoted the proliferation of breast cancer cells [15]. Additionally, knockout of c-Cbl can inhibit the proliferation of prostate cancer cells [24]. The role of Cbl-b in pancreatic cancer has not been reported. Our study showed that knockdown of Cbl-b significantly decreased the proliferative activity of PDAC cells. Moreover, in PDAC tissue samples, the expression of Cbl-b was positively correlated with the expression of Ki-67 protein, thus we suggest that Cbl-b is associated with the proliferative potential of PDAC cells.

To identify the mechanism underlying the miR-891b/Cbl-b pathway, we found that miR-891b overexpression or Cbl-b knockdown activated the Smad3/p21 signaling through the autocrine effect of TGF-β1 secreted by PDAC cell lines. The TGF-β/Smads signaling pathway has played a complicated role during tumor progression [25]. TGF-β1 has an inhibitory effect during the first stage of tumorigenesis. But in certain late-stage tumor cells escape this growth inhibition effect. It is now well established that the binding of TGF-β1 to its type II receptor can activate the TGF-β receptor type I-kinase, resulting in the phosphorylation of Smad2 and Smad3. Subsequently, phosphorylated Smad2 and Smad3 bind to the common Smad4 and form the Smad complex, which translocates into the nucleus to regulate target gene transcription, such as p21^Cip1^ [26]. In the present study, we found that miR-891b overexpression or Cbl-b knockdown increased the TGF-β1 levels in supernatants of PDAC cells. The results indicate that the up-regutation of Smad3/p21 signaling by miR-891b overexpression or Cbl-b knockdown, could be due to the autocrine effect of TGF-β1 secreted by PDAC cells. Although the regulating mechanism still remains unclear, it was reported that down-regulation of Cbl-b led to stronger activation of p38 mitogen activated protein kinase (p38 MAPK) in breast cancer cells [27]; p38 MAPK inhibitor could suppressed the up-regulation the expression of TGF-β1 induced by angiotensin II in cultured adult atrial fibroblasts [28]. Thus, our results suggest that the increased expression of TGF-β1 induced by miR-891b overexpression or Cbl-b knockdown might be mediated by p38 MAPK phosphorylation. However, this hypothesis is needed the further investigations. In addition, we found that miR-891b overexpression or Cbl-b knockdown increased the expression of Smad3 protein. In choriocarcinoma cells, the expression of Smad3 could be promoted by TGF-β1, and suppressed by p38 MAPK inhibitor [29]. In the present study, Smad3 mRNA level was increased by miR-891b overexpression or Cbl-b knockdown. Thus, it is suggested that the up-regulation of Smad3 protein induced by miR-891b overexpression or Cbl-b knockdown might be due to TGF-β1-induced Smad3 upregulation at mRNA level. However, the molecular mechanism awaits further investigation. Above all, we concluded that miR-891b activated the Smad3/p21 axis by directly targeting the Cbl-b gene to inhibit the proliferative ability of PDAC cells.

In the immune system, Cbl-b can inhibit the activation of T CD8+ cells and the immune killing ability of natural killer (NK) cells to inhibit the inherent anti-tumor immune function of the human body and accelerate the development and progression of tumors [30]. The genetic deletion or functional inactivation of Cbl-b in NK cells can significantly inhibit the proliferation and metastasis of melanomas [31]. The function of Cbl-b in tumor cells, however, is controversial. As being a negative regulator of growth factor receptor signaling and involved in the suppression of cancer cell proliferation [32], Cbl-b is regarded as having anti-tumor activity. 32D/EGFR cells overexpressing Cbl-b showed markedly reduced proliferative response to EGF, and increased the number of cells undergoing apoptosis [32]. In gastric cancer cells, Cbl-b could inhibit the survival signal of EGFR pathway to inhibit the proliferation induced by 5-FU treatment [33]. In contrast, studies have also indicated that Cbl-b promotes the proliferation of cancer cells. For breast cancer, Cbl-b binds to Smad3 through its proline-rich region and prevents the protein from transferring into the nucleus to inhibit the transcription of tumor suppressor genes downstream of the TGF-β pathway, including p21^Cip1^ and p15^INK4b^ [15]. In pancreatic cancer cells, our study showed that silencing of Cbl-b expression inhibited proliferation in PDAC cells by up-regulation of the Smad3/p21 signaling. Therefore, the effects of Cbl-b on the proliferation of different cancer cells are absolutely adverse, which may be due to the various proteins that interact with Cbl-b in different cancer cells.

Lastly, inevitable limitations exist in the present study. As shown by bioinformatics tools, a large number of potential target genes of miR-891b were identified. We couldn't validate all of these candidates in one study. Here, we have shown that miR-891b suppressed growth of pancreatic cancer by targeting Cbl-b. In the future, more studies are required to be designed to investigate the other potential target genes of miR-891b and the associated regulatory mechanism to illustrate the complicated regulatory networks of miR-891b in pancreatic cancer.

In conclusion, miR-891b regulated the Smad3/p21 axis by directly targeting the Cbl-b gene to inhibit the proliferative ability of PDAC cells. We provide a theoretical basis for the study of miR-891b/Cbl-b in pancreatic cancer and the role of miR-891b/Cbl-b in prognostic prediction, as well as the identification of new targets for PDAC.

## MATERIALS AND METHODS

### Patients and tissue samples

From 1 January 2009 to 29 February 2011, a total of 182 consecutive patients underwent surgery for potentially resectable primary pancreatic carcinoma at the Shengjing Hospital of China Medical University. In all of the patients, the diagnosis was histologically-confirmed. Among the 182 cases, 148 had PDAC; the other 34 patients had intraductal papillary mucinous adenocarcinoma (*n* = 11), mucinous carcinoma (*n* = 8), malignant endocrine tumors (*n* = 6), adenosquamous carcinoma (*n* = 5), squamous carcinoma (*n* = 3), or acinar cell carcinoma (*n* = 1). Among the 148 patients with PDAC, five had double cancer with a life-threatening phenotype (gastric stromal tumor, renal clear cell carcinoma, adrenocortical adenocarcinoma, gallbladder adenocarcinoma, and ovarian mucinous cystadenocarcinoma), all of which were subsequently excluded from our analysis. Among the remaining 143 patients with PDAC, six died in the hospital within 30 days after surgery, thus these patients were also excluded from our analysis. Of the remaining 137 patients with PDAC, the clinicopathologic characteristics and prognosis were available for 134 patients. Ultimately, 134 patients were included in the present study (Figure 1A). The patients had not received chemotherapy or radiation therapy prior to surgery. All the records of the patients were retrospectively reviewed after approval from the Research Ethics Committee of Shengjing Hospital of China Medical University.

The variables included the following: age; gender; location of the tumor; type of resection; maximal tumor diameter; histologic differentiation; margin status; tumor stage; node stage; vessel invasion; vascular tumor thrombus; invasion of adjacent organs; TNM stage; and pre-operative serum CA19-9 level. The maximal tumor diameter was defined on pathologic analysis. The margins included pancreatic resection, biliary, posterior, retroperitoneal, and mesenteric margins. The adjacent structures and organs included the common bile duct, duodenum, stomach, colon, jejunum, and spleen. The tumors were staged according to the 7th American Joint Committee on Cancer (AJCC) tumor-node-metastasis (TNM) system. The patients, or the relatives and clinicians of the patients were contacted by telephone and interviewed for patient survival or the documented day of death. The final survival data was collected on 31 December 2014. Death was verified from the official death certificates provided by local government or medical agency, or information obtained from the family members. All the records were independently reviewed by a physician to confirm the cause of death. The adjuvant chemotherapy following the surgery (intravenous gemcitabine at a conventional dose and schedule) was administered to the patients who were willing to receive, regardless of the margin status or tumor stage [5].

Among the 134 cases, 20 cases with similar clinicopathologic features and treatment but considerable different outcomes, composed the initial screening cohort for the miRNA microarray. What's more, half of the patients had an extremely poor prognosis dying within the first year of diagnosis and were classified as “poor prognosis”, and the other 10 cases survived more than 21 months, which were classified as “good prognosis”. The remaining 114 cases that were diagnosed and treated in the same period composed the evaluation cohort. The clinicopathologic characteristics of the patients for microRNA microarray or evaluation were shown in Table 1.

### RNA isolation

Total RNA was extracted from culture cells using the RNeasy mini-kit (Qiagen, Carlsbad, CA, USA) and from each FFPE PDAC tissues with a miRNeasy FFPE kit (Qiagen, USA), following the manufacturer's instructions. The extracted RNA was quantified by absorbance at 260 nm and the purity was evaluated by the absorbance ratio at 260/280 nm with a NanoDrop ND-100 spectrophotometer (NanoDrop Technologies, Rockland, DE, USA).

### MicroRNA microarray

The expression levels of 1571 human microRNAs were quantified using a GenoSensor's GenoExplorer^TM^ microRNA microarray (Tempe, AZ, USA). The hybridized miRNA chips were scanned and analyzed using an Axon GenePix 4000B scanner and GenePix Pro software (Molecular Devices, CA, USA).

### Reverse transcription-quantitative real-time polymerase chain reaction (qRT-PCR)

Total RNA was extracted as described above. The One Step PrimeScript^®^ miRNA cDNA Synthesis kit (Takara, Japan) or the PrimeScript^®^ RT reagent Kit with gDNA Eraser (Takara, Japan) was used for miRNA or mRNA reverse transcription, respectively. Relative expression of miRNA or mRNA was calculated via the comparative cycle threshold (Ct) method, and the expression of U6 small nuclear RNA or 18S ribosomal RNA was used as the reference, respectively. The sequence-specific forward primer was as follows: miR-891b, 5′-CCGCTTCCAGAGTCATTGAAAA-3′; U6, forward, 5′-GCTTCGGCAGCACATATACTAAAAT-3′, and reverse, 5′-CGCTTCACGAATTTGCGTGTCAT-3′; Smad3, forward, 5′-CGCAGGTTCTCCAAACCTAT-3′, and reverse, 5′- CGCTGGTTCAGCTCGTAGTA-3′; 18S, forward, 5′-CCCGGGGAGGTAGTGACGAAAAAT-3′, and reverse, 5′-CGCCCGCCCGCTCCCAAGAT-3′. Uni-miR qPCR Primer was included in the kit. SYBR^®^ Premix Ex Taq™ II (Perfect Real Time, Takara) or SYBR^®^ Premix Ex Taq™ II (Tli RNaseH Plus, Takara) was used for monitoring the amount of miRNA or mRNA, respectively. The PCR conditions were 30 s at 95°C, followed by 45 cycles at 95°C for 5 s, and 59°C for 34 s for miRNA, and 30 s at 95°C, followed by 45 cycles at 95°C for 5 s, and 60°C for 34 s for mRNA, using the Applied Biosystems 7500 Thermocycler (Forster, California, USA). Data were analyzed using the Applied Biosystems 7500 software program (version 2.3) with the automatic Ct setting for adapting baseline and threshold for Ct determination. qRT-PCR assays were performed in triplicate with each cDNA sample. The threshold cycle and 2^−ΔΔCt^ method were used for calculating the relative amount of the target gene.

### Cell culture

Human PDAC cell lines (SW1990 and PANC-1), colorectal cancer cell lines (HCT116 and RKO), and gastric cancer cell lines (SGC-7901 and MGC-823) were obtained from the Type Culture Collection of the Chinese Academy of Sciences (China). The cells were maintained in RPMI-1640 medium (Gibco) with 10% heat-inactivated fetal bovine serum, penicillin (100 U/mL), and streptomycin (100 mg/mL) in an atmosphere of 95% air and 5% CO2 at 37°C. The cells were sub-cultured every 2–3 days and harvested in the logarithmic phase of growth.

### Transient transfection

Transfection of plasmids, miRNA mimic, and siRNAs into PDAC cells was performed using Lipofectamine 2000 reagent (Invitrogen, Carlsbad, CA, USA) following the manufacturer's instructions. For transient transfection, cells were transfected with plasmids, miRNA mimic or siRNAs at different doses at the indicated times before functional assays were performed. The siRNAs sequences (Genepharma, Shanghai, China) for Cbl-b and Smad3 were 5′- GGAUGUGUUUGGGACUAAUtt-3′, and 5′-GCGUGAAUCCCUACCACUAtt-3′, respectively. qRT-PCR or Western blot analysis was selected to verify transfection efficiency.

### Cell proliferation assay

To assess the effects of miR-891b or Cbl-b on cell growth, SW 1990 and PANC-1 PDAC cells were seeded in six-well plates (3.0 × 10^5^ cells per well) in triplicate. The following day, cells were transfected with miR-891b mimic/non-targeting negative control mimic (NC; Ribobio, Guangzhou, China) or siCbl-b/NC. The final concentration was kept constant. SB431542 (Sigma-Aldrich, St. Louis, MO, USA), a potent inhibitor of type I TGF-β receptor, was used to test the reverse effect on the growth inhibition by miR-891b overexpression or Cbl-b knockdown. To measure the cell proliferation in culture, cells were harvested in 1 ml of medium and counted manually after 24, 48, 72, and 96 h of incubation using a hemacytometer (Hawksley, West Sussex, UK) and a bright field microscope. This was done in combination with trypan blue to stain dead cells in the culture sample.

### Reverse transcription polymerase chain reaction (RT-PCR)

The cells were cultured and harvested for the indicated times. Total RNA was extracted as described above. RT-PCR was performed with the following primer pairs for Cbl-b: forward (5′-CGCTTGACATCACTGAAGGA-3′); and reverse (5′-CTTGCCACACTCTGTGCATT-3′). GAPDH was used as a control: forward (5′-GTGGGGCGCCCC AGGCACCA-3′); and reverse (5′-CTCCTTAATGTCACG CACGATTTC-3′). PCR conditions for Cbl-b were 95°C for 5 min, 30 cycles at 95°C for 30 s, 59°C for 30 s, 72°C for 30 s, and 1 cycle at 72°C for 10 min. PCR conditions for GAPDH were 95°C for 5 min, 33 cycles at 95°C for 30 s, 56°C for 45 s, 72°C for 45 s, and 1 cycle at 72°C for 10 min. Then, the amplified products were separated on 1.5% agarose gels, and stained with ethidium bromide and visualized under UV illumination.

### Western blot assay

Standard Western blot was performed using whole cell protein lysates. Western blot analysis was performed as described in the previous study [34]. Briefly, samples were solubilized in 1% Triton lysis buffer on ice. The protein concentrations were determined using the Lowry method. Total proteins were separated by sodium dodecyll sulfate-polyacrylamide gel electrophoresis (SDS-PAGE) and electrophoretically transferred to nitrocellulose membranes (Millipore, Bedford, MA, USA). After blocking with 5% skim milk in Tris-buffered saline Tween-20 (TBST) buffer, the blots were incubated in the primary antibodies followed by secondary antibodies at the indicated time. Primary antibodies against Cbl-b (lot number: H1010, Santa Cruz Biotechnology, CA, USA), Smad3 (lot number: GR92868-1, Abcam, Cambridge, MA, USA), phospho-Smad3 (lot number: GR83035-5, Abcam, Cambridge, MA, USA), p21 (lot number: D2904, Santa Cruz Biotechnology, CA, USA), and β-actin (lot number: K0113, Santa Cruz Biotechnology, CA, USA) were used in this analysis. Proteins were detected using an enhanced chemiluminescence reagent (SuperSignal Western Pico Chemiluminescent Substrate; Pierce, USA) in the Electrophoresis Gel Imaging analysis system (DNR Bio-Imaging Systems, Jerusalem, Israel).

### Dual luciferase reporter assay

The putative miR-891b binding site in the 3′-UTR of Cbl-b was cloned downstream from a cytomegalovirus promoter-driven firefly luciferase cassette in a pMirTarget luciferase vector (OriGene, Rockville, MD, USA). The mutant constructs were generated via mutation of the complementary seed sequence to the miR-891b-binding region. SW1990 and PANC-1 cells were transiently co-transfected with reporter plasmids (500 ng), miR-891b mimic or NC (50 nmol/L), and pRL-TK (5ng) for 24 h. The dual luciferase reporter assay (Promega, Madison, WI, USA) was performed according to the manufacturer's protocol. The normalized luciferase activity was expressed as a ratio of firefly luciferase-to-Renilla luciferase units.

### Anti-tumor assays using mouse models

All *in vivo* experiments were approved by the Institutional Review Board of China Medical University. Female pathogen-free athymic BALB/c nude mice were purchased from Vitalriver (Beijing, China). SW1990 cells (1 × 10^6^) in 0.1 mL of PBS were injected subcutaneously into the right scapular region of mice. One week after the cells were injected, the mice were randomly separated into two groups, each containing three mice, and treated with miR-891b agomir or miR-NC agomir (5 nmol in 40 μl normal saline) by subcutaneous injection every 2 days. Tumors were measured with a caliper every 2 days, and tumor volume was calculated using the following formula: V = 1/2 (width × length × height). Body weights were also recorded. The tumor-bearing mice were sacrificed by cervical dislocation when the mice became moribund or on day 15, according to the protocol filed with the Guidance of Institutional Animal Care and Use Committee of China Medical University.

### Immunohistochemistry (IHC)

The 114 cases of human PDAC tissues in the evaluation cohort were used for IHC. All sections were deparaffinized in xylene and dehydrated through a graduated alcohol series followed by the standard procedure for the S-P immunohistochemical kit (Fuzhou Maixin Biological Technology Ltd., Fujian, China). Sections were incubated with anti-Cbl-b (Santa Cruz Biotechnology, CA, USA) or anti-Ki-67 (Fuzhou Maixin Biological Technology Ltd., Fujian, China) in PBS at 4°C overnight in a moist box. Immune complex visualization was performed with 3, 30-diamino-benzidine tetrahydrochloride (DAB kit; Fuzhou Maixin Biological Technology Ltd., Fujian, China). The staining was evaluated by scanning the entire tissue specimen under low magnification (× 10) and confirmed under high magnification (× 20 and × 40). The protein expression was visualized and classified based on the percentage of positive cells and the intensity of staining. Tumors with < 10% Cbl-b were regarded as negative and ≥ 10% were considered positive. Ki-67 staining was defined as weak (< 30%), moderate (30–50%), and strong (≥ 50%) expression group according to the cells with positive stained nuclei in ten high-power fields. Final scores were assigned by two independent pathologists.

### Elisa assay

The PDAC cells were transfected with miR-891b mimic/NC or siCbl-b/NC. Following 48 h of incubation, cell culture supernatants were collected and assayed with a human TGF-β1 ELISA kit (lot number: DB100B, R&D Systems, Abingdon, UK), following the manufacturer's instructions. The minimum detectable dose of human TGF-β1 ranged from 1.7-15.4 pg/mL. The mean was 4.64 pg/mL.

### Immunofluorescence

The PANC-1 cells were seeded on coverslips, which were placed in six-well plates in advance. The following day, cells were transfected with miR-891b mimic/NC or siCbl-b/NC. After incubation for 48 hours, the cells were fixed with 4% paraformaldehyde for 15 minutes, permeabilized with 0.5% Triton X-100 for 10 minutes, blocked with 1% bovine serum albumin for 1 hour and incubated with anti-Smad3 antibody at 4°C overnight. The following day, the cells were rinsed with PBS and incubated with Alexa Fluor 546-conjugated goat anti-rabbit IgG (Molecular Probes, Eugene, OR, USA) for 1 hour at room temperature in the dark. 4′, 6-Diamidino-2-phenylindole (Sigma-Aldrich) was used to stain nuclei for 5 minutes. Following mounting with the antifade mounting medium (Beyotime Institute of Biotechnology, Haimen, China), the cells were visualized by fluorescence microscopy (BX60, Olympus, Tokyo, Japan).

### Statistical analysis

Overall survival (OS) was defined as the time from the date of the surgery to the date of death or the last contact, i.e., the date of the last follow-up visit. The patients who were alive at the last follow-up evaluation were censored. Kaplan-Meier estimate was used to analyze the survival data and the statistical significance was evaluated by the log rank test. Multivariate analysis was performed using the multivariate Cox proportional hazards model (forward), which was fitted using all of the clinicopathologic variables. The comparisons between the miR-891b levels and clinicopathologic characteristics were performed with *x*^2^ test or Student's *t*-test. The Spearman correlation analysis was performed to analyze the association between miR-891b or Cbl-b levels and Ki-67 levels. The experimental data were presented as mean ± standard deviation (SD) or median (P_25_, P_75_). The differences between groups were assessed by Student's *t*-test or Mann-Whitney *U* test. All means were calculated from at least three independent experiments. Two-sided *P* values < 0.05 were considered to be statistically significant. SPSS software (version 13.0; SPSS, Inc., Chicago, IL, USA) was used for statistical analysis.

## Abbreviations

PDAC: pancreatic ductal adenocarcinoma
miR-891b: microRNA-891b
OS: overall survival
miRNA: microRNA
UTR: untranslational region
FFPEs: formalin-fixed paraffin-embedded specimens
qRT-PCR: reverse transcription-quantitative real-time polymerase chain reaction
NC: negative contorl
EGFR: epidermal growth factor receptor
siRNA: small inferfering RNA
IHC: immunohistochemistry
TKB: tyrosine kinase binding
NK: natural killer
AJCC: American Joint Committee on Cancer
TNM: tumor-node-metastasis
RT-PCR: reverse transcription polymerase chain reaction
SDS-PAGE: sodium dodecyll sulfate-polyacrylamide gel electrophoresis
TBST: tris-buffered saline Tween-20
SD: standard deviation.
